# Genetic and Cheminformatic Characterization of *Mycobacterium tuberculosis* Inhibitors Discovered in the Molecular Libraries Small Molecule Repository

**DOI:** 10.1021/acsinfecdis.4c00936

**Published:** 2025-03-25

**Authors:** Ifeanyichukwu E. Eke, John T. Williams, Robert B. Abramovitch

**Affiliations:** Department of Microbiology, Genetics & Immunology, Michigan State University, East Lansing, Michigan 48824, United States

**Keywords:** antibiotics, *Mycobacterium tuberculosis*, phenotypic screening, mechanism of action

## Abstract

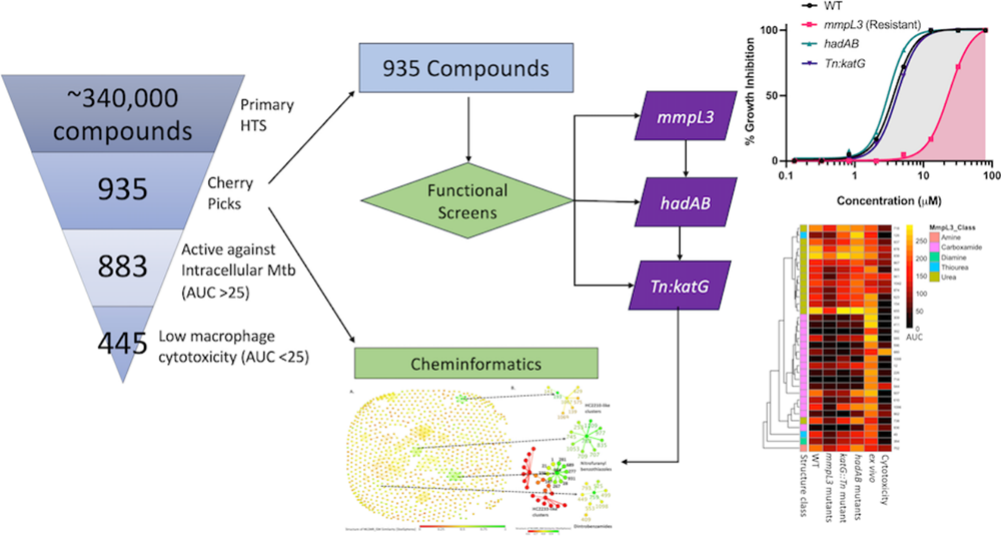

High-throughput screening (HTS) of small molecules is a starting point for many drug development pipelines, including tuberculosis. These screens often result in multiple hits whose mechanisms of action remain unknown. From our initial HTS of the Molecular Libraries Small Molecule Repository (MLSMR), we cherry-picked 935 compounds that inhibited the growth of *Mycobacterium tuberculosis* and set out to provide an early assessment of their antimycobacterial properties and mechanism of action. To characterize the MLSMR Mtb growth inhibitors, a combination of cheminformatics and targeted mutant screening against mutants in *katG*, *hadAB*, and a mixed pool of *mmpL3* mutants was used to characterize the hits. As a validation of this approach, we identified 101 isoniazid analogs that predictably lose all their antimycobacterial activities against the *katG* mutant. Interestingly, eight isoniazid analogs retain part of their activity against the mutant, suggesting an alternative KatG-independent mechanism. This approach also identified new compounds belonging to already known scaffolds that target HadAB or MmpL3. Additionally, we explored the nitro-containing compounds in our data set and discovered nitrofuranyl benzothiazoles that show enhanced activity against the *mmpL3* and *katG* mutants, a phenomenon known as collateral sensitivity. Overall, this study will serve as an important resource for further follow-up studies of antitubercular small molecules in the MLSMR library and provide a well-characterized training set for artificial intelligence-driven antimycobacterial drug discovery.

The rising incidence of drug-resistant tuberculosis (TB) demands the development of new TB drugs.^1^ Central to this effort are high-throughput screening (HTS) campaigns of different molecular libraries for agents that inhibit the growth of *Mycobacterium tuberculosis* (Mtb), followed by secondary assays to prioritize hits and mechanism-of-action studies to decipher the molecular targets of prioritized hits.

We previously conducted an HTS of a collection of ∼340,000 compounds from the National Institutes of Health Molecular Libraries Small Molecule Repository (MLSMR) to identify inhibitors of the DosRST two-component regulatory system.^2^ The MLSMR is a diverse library of chemically synthesized small molecules and natural products that were collected by the National Institutes of Health from different academic and commercial sources for distribution and testing by the biomedical community. Our initial screen of the MLSMR library was conducted using the Mtb CDC1551 (*hspX’::GFP*) reporter strain that exhibits hypoxia-inducible, DosRST-dependent fluorescence. In this screen, we identified several distinct classes of inhibitors that selectively inhibited fluorescence but not growth. These inhibitors directly targeted the DosS or DosT sensor kinases, or the DosR response regulator to inhibit the signaling pathway.^2−4^ However, the screen also identified numerous compounds that inhibited Mtb growth, presumably independent of DosRST. Since the DosRST signaling pathway is not required for growth under the screening conditions, these compounds potentially represent new Mtb growth inhibitors. Notably, a subset of these compounds was previously screened for growth inhibition of Mtb under different conditions.^5,6^

In the current study, we sought to characterize 935 Mtb growth-inhibiting compounds identified from the HTS. Cherry-picked samples of these 935 inhibitors were subjected to functional and cheminformatic characterizations and are henceforth referred to as MLSMR Mtb inhibitors. The compounds were functionally characterized in a series of dose–response secondary assays for *in vitro* potency against Mtb; *ex vivo* activity against Mtb in primary murine, bone marrow-derived macrophages; and cytotoxicity in macrophages. Next, we sought to characterize the mechanisms of action of the compounds. Forward genetic selection is commonly used to identify the molecular targets of antimycobacterial compounds. However, this method is limited by the slow-growing nature of Mtb, with resistant colonies taking several weeks to develop.^7^ With the large number of MLSMR Mtb inhibitors, it is laborious to use forward genetic selection to characterize their molecular targets. Previously, we successfully used a targeted mutant screening approach to identify inhibitors that target MmpL3, an essential mycobacterial protein.^8^ This method is amenable to HTS and involves the consecutive screening of prioritized hits against wild-type (WT) Mtb culture and a pooled mutant library of a specific gene. After screening, the potency of the hits against the WT and mutant library is compared. The working principle of this approach is that the mutant pool will be cross-resistant to the molecules that normally target the WT version of the protein of interest, but will retain its susceptibility against other molecules. Due to the success of this method in revalidating already known MmpL3 inhibitors and discovering new scaffolds,^8^ we extended this approach to functionally characterize the MLSMR Mtb inhibitors.

Using an 8-point dose–response, we tested the MLSMR Mtb inhibitors for activity against an *mmpL3* mutant pool, a *katG* transposon mutant, and a *hadAB* mutant pool and compared the potency of the compounds with that against the WT. The selection of these mutants is due to the essential roles they play in mycobacterial physiology and drug discovery. HadAB is a complex of HadA and HadB proteins that is involved in the FAS-II pathway of mycobacterial mycolic acid biosynthesis, serving as an essential dehydratase for a mycolic acid intermediate.^9−11^ MmpL3 is also involved in cell envelope synthesis, but its role is as a transporter where it uses energy from the proton motive force to move trehalose monomycolate across the cytoplasmic membrane for subsequent incorporation into the mycolic acid-rich outer membrane of mycobacteria.^7^ Unlike HadAB and MmpL3, KatG is not involved in mycolic acid biosynthesis or transport. Instead, it is part of the response machinery of the bacteria to oxidative stress and is the activator of the first-line TB prodrug, isoniazid.^13,14^ Therefore, our genetic counter-screening approach against the *mmpL3*, *hadAB*, and *katG* mutants, coupled with cheminformatic analyses, provided an early mechanism-of-action assessment of some compounds in the data set, *vis-à-vis* putative MmpL3 inhibitors, isoniazid-like compounds, and putative HadAB inhibitors. We also identified compounds that exhibited enhanced activity against the mutants, which is a phenomenon known as collateral sensitivity. Lastly, given their proven utility as TB drugs, we provide detailed analyses of some nitro-containing scaffolds in the data set. Overall, this study will serve as an important resource for further prioritization and follow-up studies of the MLSMR Mtb inhibitors. Additionally, this well-characterized resource should prove to be useful as a training set for artificial intelligence-driven antimycobacterial drug discovery.

## Results and Discussion

### *In Vitro* and *Ex Vivo* Efficacy of the MLSMR Mtb Inhibitors and Eukaryotic Cytotoxicity

Our previous single-dose HTS of the ∼340,000-compound NIH’s MLSMR library resulted in about 15,000 compounds that showed >50% inhibition of the growth of Mtb.^2^ Full screening results are publicly available in the PubChem database (BioAssay AID: 1159583). From these ∼15,000 growth inhibitors, we cherry-picked 935 compounds and henceforth referred to them as the MLSMR Mtb inhibitors. Cherry-picked compounds were selected based on chemical diversity, strength of growth inhibition, and limiting compounds with structural alerts. Structures, chemical properties, and primary HTS data are provided in Database 1, which can be browsed using the freely available DataWarrior software.^15^ To browse the structures, simply open the Database_1.txt file provided in DataWarrior. To confirm the efficacy of these compounds, we examined Mtb growth inhibition using an 8-point dose–response study, against extracellular and Mtb growing *ex vivo* in infected primary murine bone marrow-derived macrophages. The growth inhibition values of some of the compounds could not be fitted into the four-parameter logistic equation that is normally used in calculating the half-maximal effective concentration (EC_50_); therefore, we opted to use the area under the curve (AUC) as a relative measure of the potency of the compounds. We had previously used this approach to compare the potency of MmpL3 inhibitors against WT and *mmpL3*-resistant mutant pool, with the MmpL3 inhibitors having a large AUC when tested against the WT and a smaller AUC against the mutant pool.^8^ Therefore, it follows that compounds with strong potencies normally give rise to large AUC values. To assist in relating the AUC of dose–response curves, we calculated EC_50_ and MIC values for eight hypothetical dose–response curves covering AUCs from 0 to ∼280 (Figure S1). However, care should be taken with this interpretation since the AUC is only a relative measure of potency and is mostly robust for a single compound that is tested across multiple identical backgrounds^16^—the core of the experimental design of our current study.

**Figure 1:**
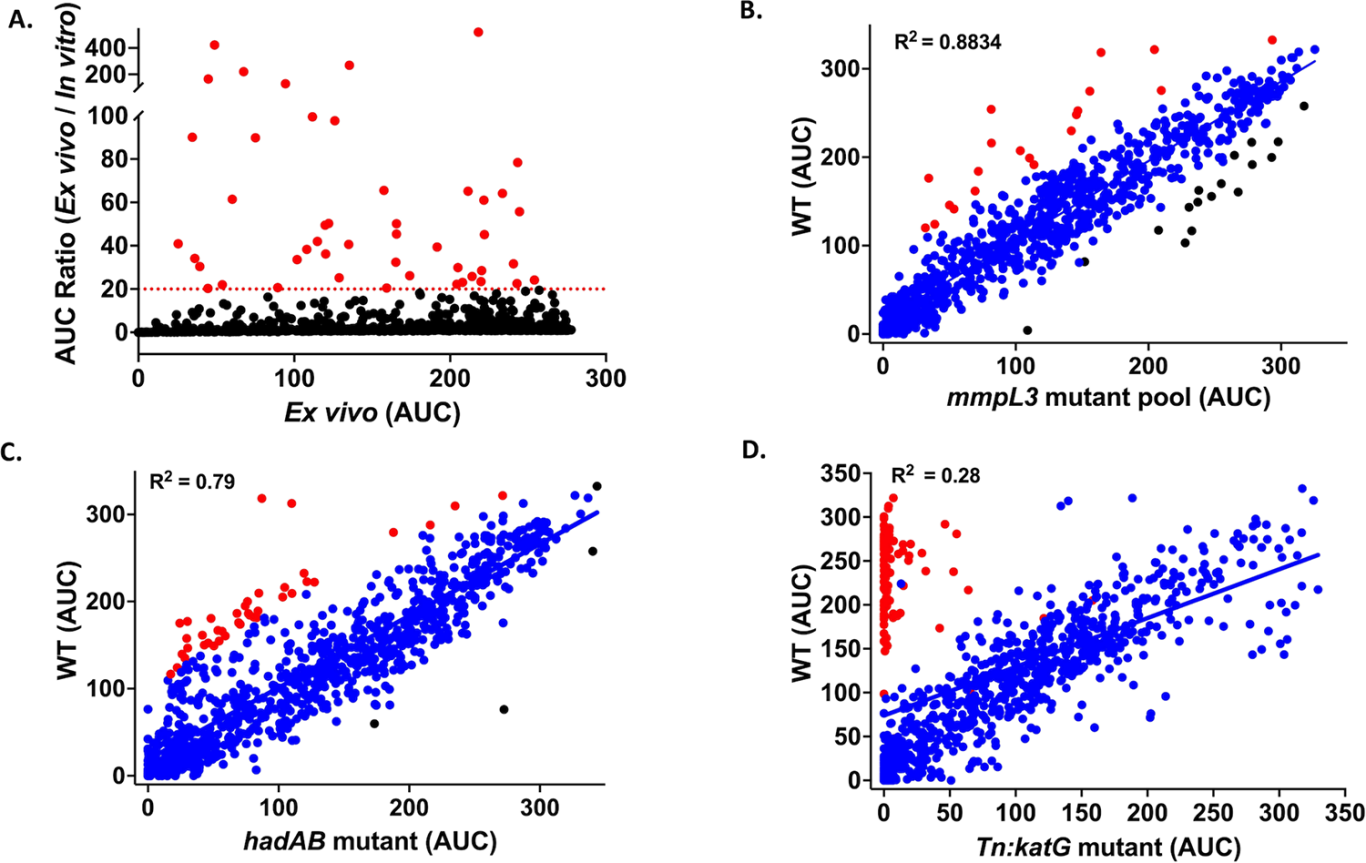
Targeted high throughput screening of the MLSMR data set. (A) Identification of compounds from the MLSMR data set that have more *ex vivo* activity compared to *in vitro* activity. The AUC ratio for each compound was calculated by dividing its *ex vivo* AUC value by the *in vitro* AUC value. Those that do not cross the 20 AUC ratio cutoff are represented in black, while red indicates those that crossed the cutoff. Eleven compounds are not represented here since their AUC ratio cannot be calculated (*in vitro* AUC is 0), but they can be seen in Data set 1. (B) Comparison of the activity of the compounds in the MLSMR data set against the WT and *mmpL3* mutant pool. MmpL3 inhibitors are marked in red from the Mahalanobis outlier analysis, and hits that show enhanced activity against the mutant are represented in black (*p* value < 0.05). The rest of the MLSMR data set are in blue. (C) Comparison of the activity of the compounds in the MLSMR data set against the WT and *hadAB* mutant. HadAB inhibitors are marked in red from the Mahalanobis outlier analysis, and hits that show enhanced activity against the mutant are represented in black (*p* value < 0.05). The rest of the MLSMR data set are in blue. (D) Comparison of the activity of the compounds in the MLSMR data set against the WT and *Tn:KatG* mutant. Those in red are isoniazid analogs, while blue represents other compounds in the MLSMR data set.

Using an arbitrary AUC cutoff of 25 for classification, 81.4% (*n* = 761) of the MLSMR Mtb inhibitor cherry-picks were confirmed as growth inhibitors of extracellular Mtb (Data set 1). This high confirmation rate is consistent with the high Z-factor (0.9) of primary HTS.^2^ When we analyzed for the inhibition of intracellular Mtb in primary murine bone marrow-derived macrophages, 94.4% (*n* = 883) crossed the 25 AUC cutoff (Data set 1), suggesting that some compounds may have higher activity in macrophages as compared to *in vitro*. To highlight some of these compounds, we divided the *ex vivo* AUC of each of the compounds by their *in vitro* values and plotted these ratios against the *ex vivo* AUC values (Figure 1A, Data set 1). This approach identified 58 compounds that showed higher activity against intracellular Mtb. Examination of the structures of some of these compounds showed the presence of groups that are known for their bias toward a higher intracellular activity. These include the thiophene carboxamides, which have been previously reported by Ahmed and colleagues^38^ to have growth inhibitory effects against intracellular Mtb but lose these effects against the extracellular pathogen. Out of the 58 compounds with higher *ex vivo* activity in our study, we could count at least 7 compounds that possess a thiophene carboxamide scaffold (**104**, **774**, **30**, **1082**, **1094**, and **380**). While this confirms the validity of our approach in identifying compounds with higher *ex vivo* activity, follow-up studies need to be done, especially for compounds that occurred more than once on the screen. Next, we tested for the eukaryotic cytotoxicity of the MLSMR data set in bone marrow-derived macrophages. About 47.6% (*n* = 445) of the tested compounds did not cross the 25 AUC cutoff, indicating limited cytotoxicity. Additionally, when we calculated the selectivity index of the compounds using the AUC values, we observed that the index values of most of the compounds were greater than one (*n* = 812), indicating higher *ex vivo* activity compared to eukaryotic cytotoxicity (Data set 1). Additionally, when we used a selectivity index cutoff of 10 that is normally considered favorable in antimicrobial drug development, we observed 373 compounds that crossed the selectivity index of 10 and these include compounds whose selectivity indexes could not be calculated since they showed they did not have any cytotoxicity (AUC = 0) but had varying *ex vivo* antimycobacterial activity (Data set 1).

### Targeted Mutant Screening and Analyses

To decipher the biological activity of the MLSMR Mtb inhibitors, we screened the compounds in a dose–response against an *mmpL3* mutant pool (composed of 24 separate *mmpL3* mutants),^8^ a *katG* transposon mutant that is resistant to isoniazid, and a *hadAB* mutant pool (composed of 3 *hadA* and *hadB* mutants). By comparing the potency of the compounds against the transposon mutant or mutant pools with the WT (Data sets 2–4, Figure 1), we identified compounds with significantly decreased or enhanced potency, and we set out to analyze the individual screens.

### Putative MmpL3 Inhibitors

**Figure 2:**
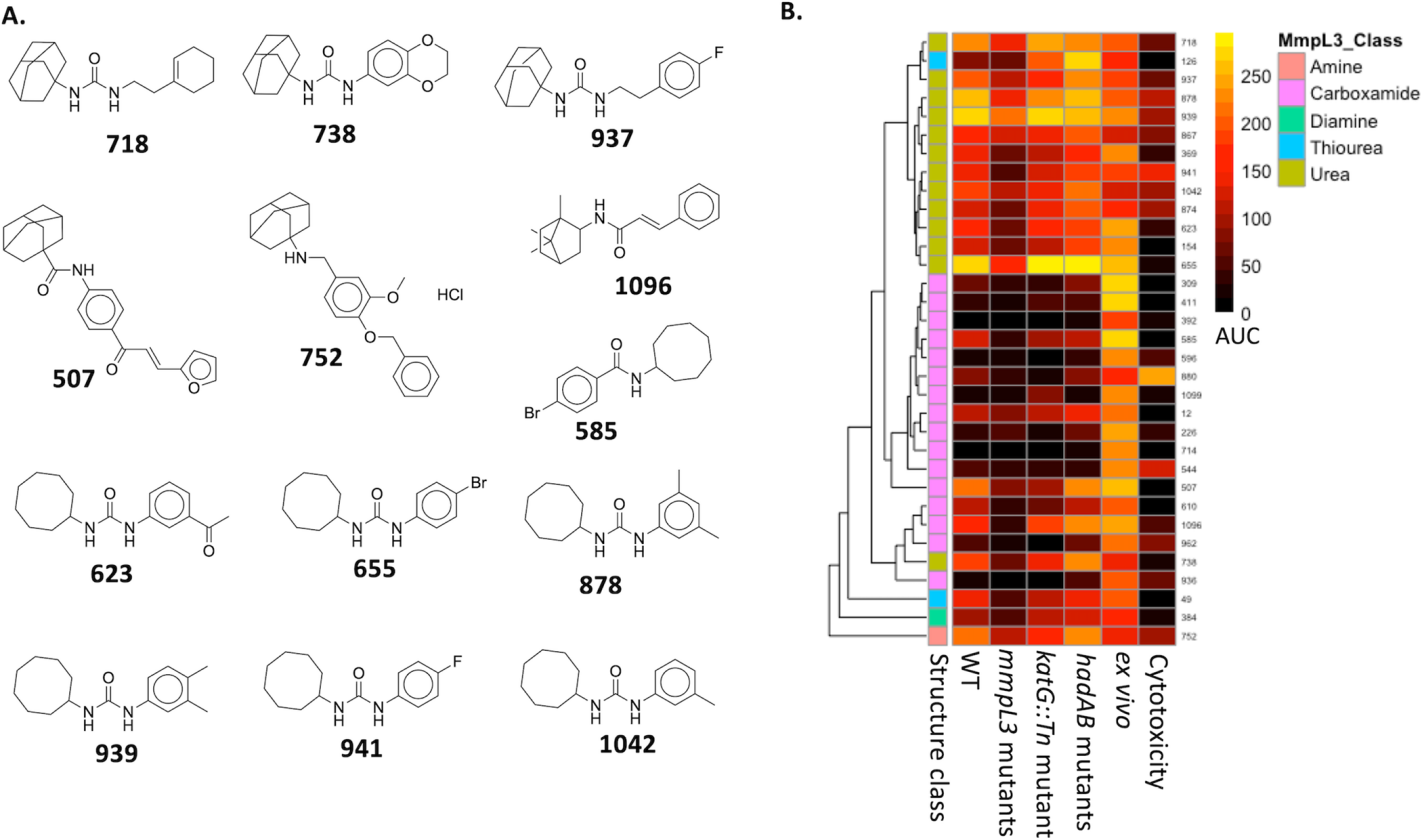
Identification of putative MmpL3 inhibitors from the *mmpL3* mutant screen. (A) Representative hits identified from the outlier analysis of the *mmpL3* mutant scatterplot. (B) Hierarchical dendrogram of the discussed putative MmpL3 inhibitors.

In the *mmpL3* mutant screen, we observed a strong correlation in the activity of the MLSMR hits against the WT and the *mmpL3* mutant pool (*R*^2^ = 0.89), and we used the Mahalanobis distance method to identify significant outliers in the scatterplot (Figure 1B). At a significance threshold of *p* value < 0.05, 37 compounds were identified as outliers in the screen (Data set 2). Out of these outliers, 20 compounds showed a significant loss of activity against the *mmpL3* mutant pool and were classified as putative MmpL3 inhibitors (Data set 2, Figure 2). Among these 20 putative MmpL3 inhibitors were five adamantyl-based compounds, and they belonged to different classes such as adamantyl ureas (**718**, **738**, and **937**), adamantyl carboxamide (**507**), and adamantyl amine (**752**) compounds. This is in line with numerous studies that have genetically and biochemically confirmed these adamantyl-based scaffolds as MmpL3 inhibitors.^7,8,17−19^ To identify other adamantyl-containing compounds in our data set, we did a substructure similarity search with adamantyl as the query substructure. This gave rise to 14 additional adamantyl-based analogs (Data set 2). However, two of these compounds contained an isoniazid backbone (**863** and **961**) and will be discussed in another section of this paper. Some of these adamantyl-based compounds that were identified from our query search showed reduced activity against the *mmpL3* mutant and were missed by our stringent outlier cutoff. They include **384**, **496**, **544**, **610**, and **912**, among others. Interestingly, **126**, an adamantyl thiourea that was identified from the query search, had an insignificant potency loss against the *mmpL3* pool but showed enhanced antimycobacterial activity against the *hadAB* and *Tn:katG* mutants. This may be an example of collateral sensitivity, where a compound shows enhanced activity against resistant mutants,^39^ although additional studies are required to confirm this observation.

Consistent with a previous study from our lab,^8^ our outlier analysis also identified **1096**, a bicycloheptanyl carboxamide, as a putative MmpL3 inhibitor (Data set 2, Figure 2). Using bicycloheptanyl carboxamide as a query in a substructure similarity search, we identified other bicycloheptanyls in our data set (Data set 2), including those linked to a dinitrobenzamide (**641**), isoniazid (**422**), or a fluoroquinolone (**907**). As expected, **641**, **422**, and **907** are highly potent and retain their activity against the *mmpL3* mutant pool, while all other bicycloheptanyl carboxamides in our data set lost their activity against the *mmpL3* mutant (Data set 2, Figure 2).

In addition to the five adamantyl-based compounds and the bicycloheptanyl carboxamide, our statistical outlier analysis of the *mmpL3* mutant screening data identified one cyclooctyl carboxamide (**585**) and six cyclooctyl ureas (**623**, **655**, **878**, **939**, **941**, and **1042**) as potential MmpL3 inhibitors (Data set 2, Figure 2). Notably, our lab has previously characterized a cyclooctyl piperazine (HC2178) and a cyclohexyl urea (HC2138) as MmpL3 inhibitors.^8^ As a complementary approach, we queried the MLSMR data set for other cyclooctyl-based compounds and were able to identify putative MmpL3 inhibitors (Data set 2, Figure 2) such as **12**, **154**, **867**, and **874**. Our outlier analysis also showed that a cyclohexyl amine (**862**) and a cyclopropyl urea (**673**) significantly lose their antimycobacterial activity against the *mmpL3* mutant (Data set 2, Figure 2). Overall, these compounds represent new additions to the increasing portfolio of MmpL3 inhibitors and need to be further studied.

To provide a holistic picture and increase the robustness of our analyses, we collected all the MmpL3 inhibitors that were identified from the outlier method or chemical similarity search (about 35 compounds) and generated a hierarchical dendrogram that is based on their chemical similarity paired with their activities in each assay described above. This clustering method demonstrated that MmpL3 inhibitors clustered based on their central core groups^7,20^ and were distinguished between amines, carboxamides, diamines, thioureas, and urea-based MmpL3 inhibitors (Figure 2B).

### Thiosemicarbazones and Other Putative HadAB Inhibitors

**Figure 3:**
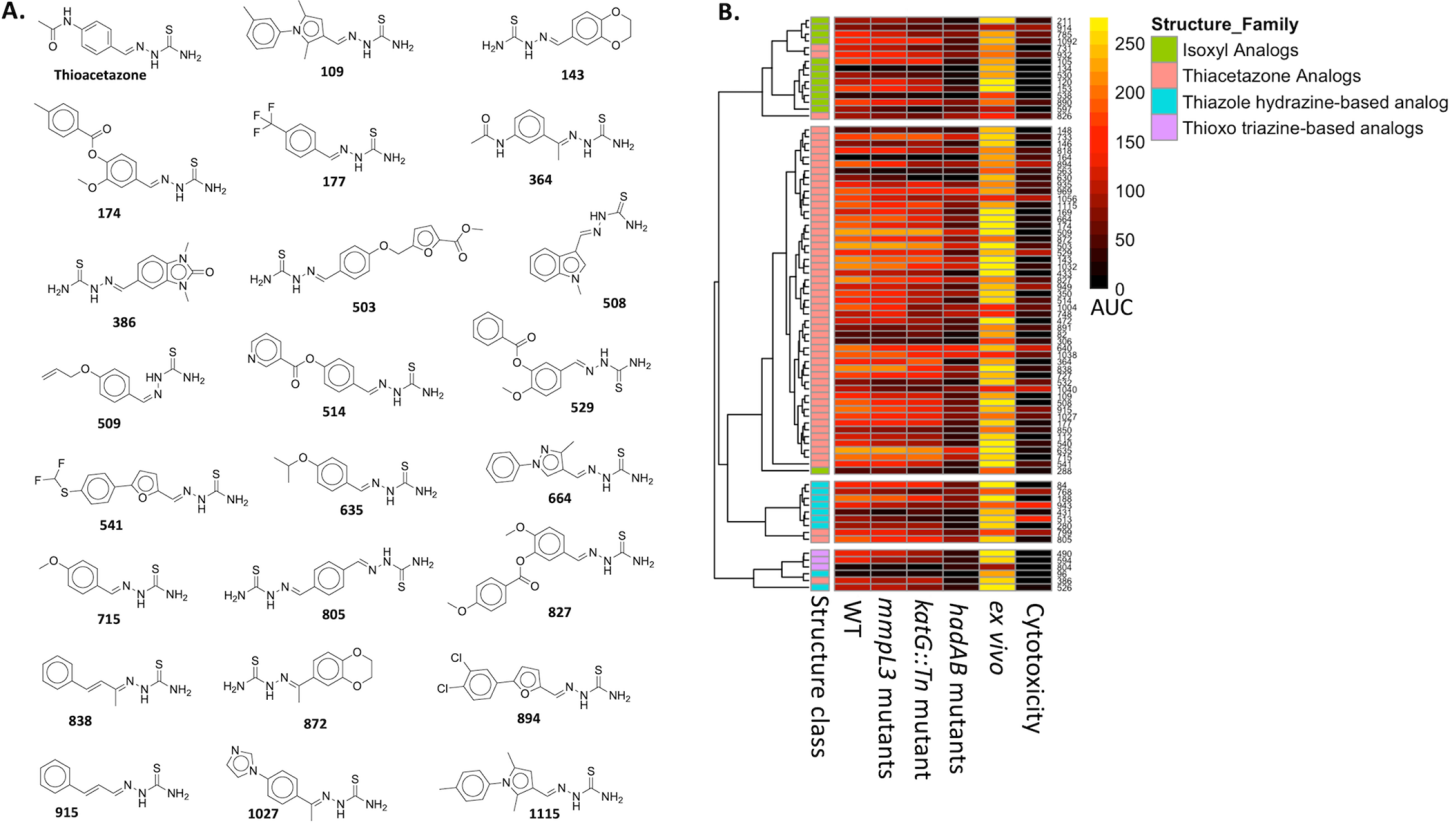
Identification of putative HadAB inhibitors from the *hadAB* mutant screen. (A) Thiosemicarbazone-based compounds identified from the outlier analysis of the *hadAB* mutant screen. The structure of the antitubercular drug, thiocetazone, is included here for comparison. (B) Hierarchical dendrogram of the discussed putative HadAB inhibitors.

To analyze the *hadAB* mutant screen, we generated a scatterplot of the activities of the MLSMR data set against the WT and *hadAB* mutant pool and showed that the data set had a strong correlation between the two variables (*R*^2^ = 0.79) (Figure 1C), and we used the Mahalanobis method as before to identify outliers. This analysis identified 44 outliers, with 40 classified as putative HadAB inhibitors based on their potency loss against the mutant pool (Data set 3). Interestingly, 23 of these putative HadAB inhibitors were thioacetazone (TAC)-based compounds (Data set 3, Figure 3). This was not surprising since TAC is a well-known thiosemicarbazone-based bacteriostatic prodrug that targets the HadAB or HadBC dehydratase complex of the FAS-II pathway.^9−11^ The activating mycobacterial protein for TAC is EthA, an FAD-containing monooxygenase, and this protein also serves as the activator for ethionamide.^21^ When activated, both TAC and ethionamide inhibit mycolic acid biosynthesis, targeting different proteins that are involved in the FAS-II pathway of mycolic acid biosynthesis. Ethionamide shares the same target with isoniazid, both targeting InhA of the FAS-II pathway, while, as noted previously, TAC targets the HadABC component of the pathway.

Reasoning that there might be other thiosemicarbazone-based compounds that have been overlooked by our stringent statistical approach, we used thiosemicarbazone as a query in a substructure similarity search of the MLSMR data set. This resulted in an additional 33 thiosemicarbazone-containing compounds, with all of them showing reduced activity against the *hadAB* mutants (Data set 3, Figure 3). Together, our data suggest these thiosemicarbazones as putative HadAB inhibitors, although further validation is needed.

We also identified two thiazole hydrazine-based compounds (**84** and **188**) as outliers in the *hadAB*-resistant mutant screen (Data set 3, Figure 3). A substructure similarity search of the MLSMR data set revealed seven more thiazole hydrazine-based compounds that might be putative HadAB inhibitors (Data set 3, Figure 3). Additionally, **490**, a thioxotriazine, came out from our outlier analysis as a novel scaffold with less activity in the *hadAB* mutant (Data set 3, Figure 3). There are two other thioxotriazines in our data set, but only one (**594**) showed reduced activity against the *hadAB* mutant (Data set 3, Figure 3).

In addition to TAC, isoxyl (ISO), a 1,3-diphenylthiourea-based compound, is known to target the HadAB and HadBC complexes.^22^ Indeed, our outlier analysis identified four ISO analogs (**105**, **153**, **890**, and **1092**) as having lower activity in the *hadAB* mutant pool (Data set 3, Figure 3). Based on this, we hypothesized that additional ISO analogs were present in our compound library. To test this, we performed a substructure chemical search for 1,3-diphenylthioureas to identify additional ISO analogs. The result of this search identified nine additional ISO analogs (**120**, **134**, **211**, **288**, **530**, **538**, **597**, **785**, and **914**). While compounds **120**, **211**, **288**, **530**, **538**, **597**, and **785** all demonstrated lower activity in the *hadAB* mutant pool compared to the WT, **134** and **914** did not show any reduced activity against the mutant.

Clustering analysis was undertaken to categorize *hadAB* inhibitors based on their inhibitory activities and chemical structures. In the resulting dendrogram, distinct clusters/scaffolds can be seen, including isoxyls, thiacetazones, thiazoles, and thioxos (Figure 3). This clustering reflects the broad structural diversity of the compounds that potentially target the HadAB complex. The dendrogram also showed that these compounds retained modest activity in extracellular culture (median AUC = 137.5), but had a more pronounced activity against intracellular Mtb (median AUC = 240). They also had lower cytotoxicity effects against the bone marrow-derived macrophages, further buttressing their potential development as TB drugs.

### Isoniazid and Isoniazid-Based Compounds

**Figure 4:**
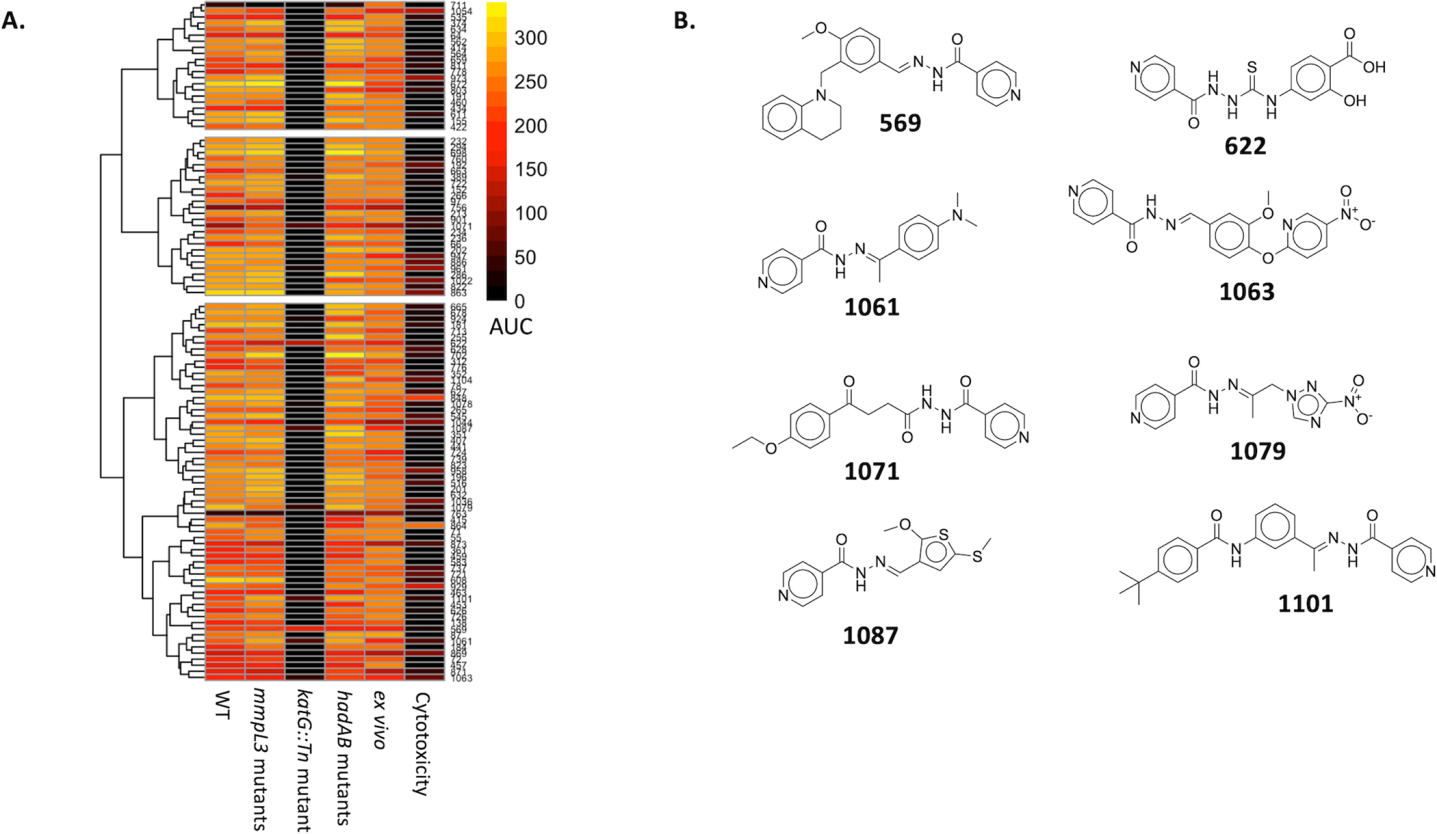
Analysis of the isoniazid analogs that were identified from the *katG Tn* mutant screen. (A) Clustering of the 109 isoniazid analogs in the MLSMR data set. (B) Structures of the isoniazid analogs in the MLSMR data set that do not completely lose their activity against the *katG Tn* mutant (they have an AUC of >40 against the *katG Tn* mutant)*.*

We could not use the Mahalanobis outlier method to analyze the *katG* mutant screen since there was a poor correlation in the resulting scatterplot of the activities of the MLSMR data set against the *katG* Tn mutant and WT (*R*^2^ = 0.28) (Figure 1D). However, there was a distinct cluster of compounds in the scatterplot that completely lost their potency against the *katG Tn* mutant (Figure 1D). We hypothesized that these compounds are enriched in isoniazid analogs since isoniazid is a prodrug that depends on KatG for activation into an antimycobacterial metabolite.^13,14^ It follows that without a functional *katG* gene isoniazid will not inhibit the growth of Mtb. Indeed, a substructure similarity search identified 109 isoniazid analogs (Data set 4, Figure 4), with most of them (*n* = 101) completely losing their activity against the *katG Tn* mutant. Interestingly, some isoniazid analogs (*n* = 8) retain partial antimycobacterial activity against the transposon mutant (Figure 4B), suggesting an additional KatG-independent system for inhibiting the growth of Mtb. These potential multitarget compounds could be useful agents that limit the evolution of resistance. Additionally, all of the 109 isoniazid analogs retain their activities against the other tested mutants, with most of them having a low cytotoxicity profile (Figure 4).

The large number of isoniazid analogs in our data set makes it conducive for a structure–activity relationship (SAR) study. Therefore, we explored the SAR of the analogs in an activity cliff analysis. We used the Skelphere molecular descriptor of the analogs as a measure of their structural similarities and the AUC of the analogs against the WT as a measure of the activity or potency. This activity cliff analysis gave rise to defined clusters of the analogs based on their structure–activity landscape index (SALI). SALI values are calculated from the chemical similarities of compounds as well as their antimycobacterial activities.^23^ The higher the SALI value, the more significant the change in the activity of the analogs when a minor structural modification is made. As shown in Figure S2 and Data set 4, most of the analogs had a small SALI (<1000), indicating nonsignificant potency changes resulting from the structural modifications. However, there are some analogs that have large SALI values (>1000). These represent modifications that can be pursued by medicinal chemists to further optimize the analogs. As an example, we will discuss a few pairwise comparisons here (Figure S3), but all 288 pairwise comparisons that resulted from the activity cliff analysis of the isoniazid analogs can be seen in Data set 4.

Compounds **152** and **1071** have a 97% structural similarity; however, shortening the length of the alkyl group that is linked to the phenyloxy group of the latter significantly reduced its activity (Figure S3). This same pattern can also be seen for **213** and **1071**; as well as **192** and **1071**, where longer-chained alkyl groups attached to the terminal phenyl or phenyloxy groups consistently led to a higher activity against the WT. **871** and **1036** have a 96% similarity and differ only in the presence of a terminal propionamide group in the former and an acetamide group in the latter. However, **871** had substantially higher activity than **1036**, illustrating the detrimental nature of the acetamide group. Lastly, **763** and **864** are highly similar to each other (89%) and only differ based on the position of the nitro group in their shared nitrophenyl moiety. While **864** has a 2-nitrophenyl group and had a higher activity, **763** has a 3-nitrophenyl group that is antithetical to its antimycobacterial activity. This point is further illustrated in **71** and **763**; as well as **415** and **763** (Figure S3). Notably, although beyond the scope of this study, it is possible to extend this analysis from the collection of cherry-picked compounds to all of the compounds in the MLSMR collection to identify modifications impacting activity.

### Nitro-Containing Compounds

#### Nitrofuranyl Piperazine Benzene-Based Compounds

**Figure 5:**
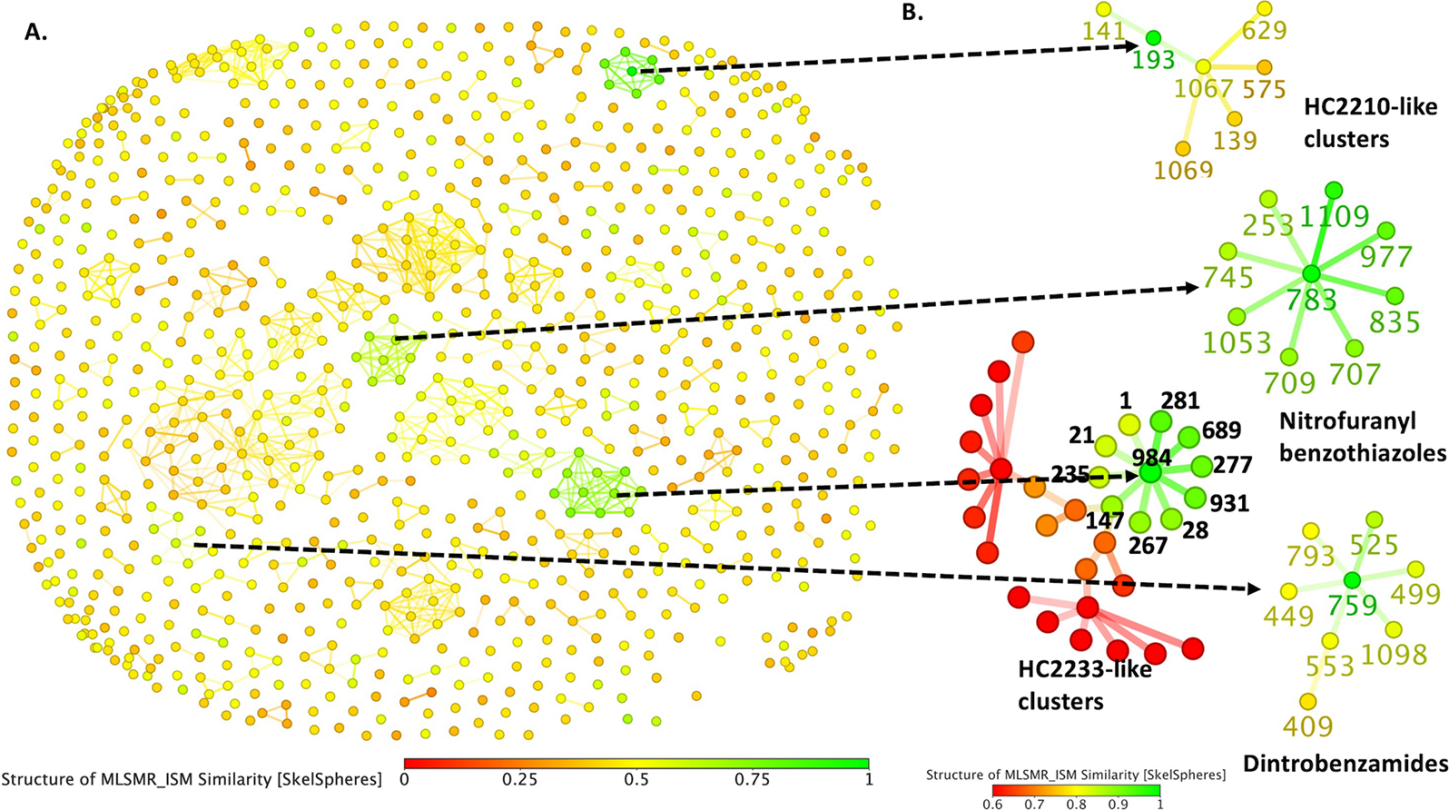
Chemical similarity clustering of all the compounds in the MLSMR data set. (A) Similarity Skelphere of the MLSMR clusters and (B) neighborhood tree visualization of different nitro scaffolds that cluster together.

In our previous study, we showed three nitrofuranyl piperazine benzene-based compounds (HC2209, HC2210, and HC2211) from the MLSMR Mtb inhibitors act as antimycobacterial prodrugs that depend on the mycobacterial deazaflavin machinery and its attendant nitroreductase(s) for activation into possible toxic metabolites.^24^ In our bid to identify other analogs in the MLSMR data set, we used the nitrofuranyl-piperazine-benzene parent structure as a query in a substructure similarity search. This analysis identified seven analogs including the already described HC2209, HC2210, and HC2211 (Data set 5). As a complementary cheminformatic approach, we clustered the whole MLSMR Mtb inhibitor collection using the Skelphere molecular descriptor. Predictably, the seven analogs clustered together, suggesting that we did not miss any related analogs in the collection (Figure 5).

**Figure 6:**
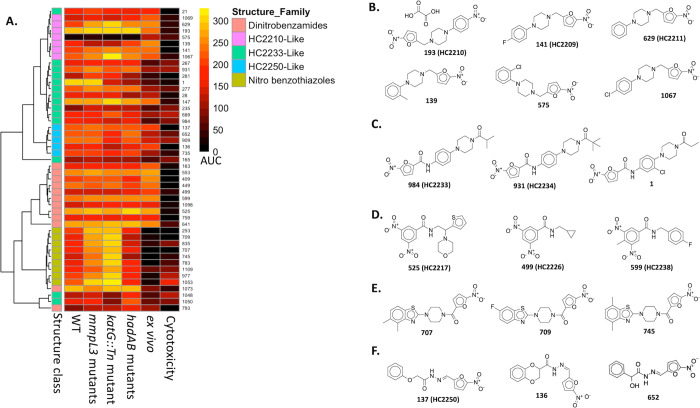
Analysis of nitro-containing compounds in the MLSMR data set. (A) Clustering of the nitro-containing compounds discussed in this paper. Structures of nitro-containing scaffolds that include representative molecules from (B) HC2210-like compounds; (C) HC2233-like compounds; (D) dinitrobenzamides; (E) nitrobenzothiazoles, and (F) HC2250-like compounds.

Next, we characterized the inhibitory activities of these analogs by comparing their potencies, as defined by the AUC, against the WT and the tested mutants (Data set 5, Figure 6). While the number of analogs is too small for a comprehensive SAR study, HC2210 is the most potent analog against the WT (AUC = 287.2). We have so far confirmed HC2210 to have a drug-like EC_50_ of 50 nm *in* vitro and to be effective in a chronic murine model of tuberculosis when delivered once daily and orally at 75 mg/kg.^24^ In contrast, **575** had the lowest potency against the WT (AUC = 28.79). This is suggestive of SAR around the phenyl group since **1067**, the closely related analog of **575**, maintained a high potency against the WT (AUC = 221.3). The two analogs differ only in the position of the substituted chlorine group in the phenyl moiety (Figure 6B). **139** and **1069** are also closely related analogs, with the latter having a dimethyl group attached to the phenyl and the former having a methyl group. These compounds have similar potencies against WT, suggesting that the methyl-based substitutions do not impact their inhibitory activities. **139** and **1069** also differ in terms of their activities against the mutants. While **139** loses some of its activities against the *mmpL3* mutant pool and the *Tn:katG* mutant, **1069** retains its activities against the mutants. HC2209, HC2210, HC2211, and **1067** showed slightly enhanced potencies against the three mutants, although further studies are needed to confirm the possibility of collateral sensitivity.

#### Nitrofuranyl Carboxamides

Following the drug discovery efforts of Lee and colleagues,^25,26^ nitrofuranyl carboxamides have emerged as important antimycobacterial compounds.^5,6,24^ Our group recently characterized two nitrofuranyl carboxamides—HC2233 (compound **984**) and HC2234 (compound **931**)—from the MLSMR Mtb inhibitors to be active against replicating and nonreplicating Mtb.^24^ These compounds are active in both *ddn* and *fgd* mutants supporting that they do not require the cofactor F_420_-dependent activation mechanism. Due to the potent activity of this series against nonreplicating persistent Mtb, we were interested in identifying other analogs in the MLSMR data set using chemical similarity clustering. This approach gave rise to 9 analogs that clustered closely with HC2233 and HC2234 (Figures 5 and 6, Data set 5). Among these series, **1**, with substituted 3-chlorophenyl and 4-propanoylpiperazine rings had the highest potency against the WT (Data set 5, Figure 6). It has two close relatives (**235** and **277**) that differ only in terms of the length of the alkyl group attached to the terminal carbonyl group (Figure 6B). **1** has an ethyl group attached to the terminal carbonyl group, while **235** has an acetyl group, and **277** has an isopropyl group. Interestingly, **1** and **277** showed similar potencies against the WT. However, **235** was different from the two compounds in terms of its lower potency against the WT, suggesting a negative impact of the substituted acetyl group on the antimycobacterial activity of the compound. Also, of interest, **1** and **281,** only differ by a chloro-group on the phenyl ring, with the chloro-substitution resulting in an almost 3-fold increase in the AUC.

Examining the activities of this series against the mutants showed that most of the analogs retained their inhibitory activities against the tested strains (Figure 6, Data set 5). Additionally, we queried the MLSMR Mtb inhibitors in a substructure similarity search for analogs of 5-nitrofuran-2-carboxamide. This uncovered 14 nitrofuranyl carboxamide-containing molecules, including the 11 benzyl piperazine/piperidine-linked molecules that clustered closely with HC2233 and HC2234 (Figures 5 and 6, Data set 5). The other three compounds are either conjugated with an oxadiazole phenyl group (**1048** and **1050**) or a phenyl carboxamide furanyl group (**165**).

#### Dinitrobenzamides

We have recently characterized four dinitrobenzamides (HC2217, HC2226, HC2238, and HC2239) from the MLSMR Mtb inhibitors as putative DprE1 inhibitors.^24^ This is in line with previous studies that have genetically and biochemically established dinitrobenzamides as DprE1 inhibitors.^27,28^ To uncover other dinitrobenzamides in the MLSMR data set, we carried out a substructure similarity query of the data set. This resulted in 12 dinitrobenzamide analogs, including the already described four compounds (Data set 5). Structural similarity clustering showed that eight of the identified analogs clustered together (Figure 5), while the other four compounds are found either in singletons or pairs. A look at the activity of the compounds against the WT showed that they maintained a relatively high potency against the WT, although **793** and **499** exhibited moderate activity (Figure 6). When we extended the investigation to the mutant strains, we also observed that all the dinitrobenzamides maintained their activities against the mutants. This is predictable since the target proteins are involved in synthesizing different components of the cell wall. DprE1 is involved in the synthesis of arabinogalactan, while MmpL3 and HadAB are catalyzing different steps in mycolic acid synthesis. Additionally, dinitrobenzamides are mechanism-based DprE1 inhibitors and do not primarily work through the production of reactive oxygen species. Thus, disruption of the *katG* gene should not have any effect on the activity of the compounds.

#### Nitrofuranyl Hydrazides

Recent work by Batt and co-workers^29^ identified two 5-nitrofuran-2-carbohydrazides as DprE2 inhibitors that possibly depend on the deazaflavin system for activation into active metabolites. This report was closely followed by ours, which characterized a 5-nitrofuran methylidene hydrazide (HC2250) from the MLSMR data set as a putative DprE1 inhibitor.^24^ However, we showed that HC2250 does not depend on the deazaflavin activation machinery. Together, these two reports represent characterizations of nitrofurans as inhibitors of the DprE1/E2 complex. To uncover other putative DprE1/E2-targeting nitrofuranyl hydrazide analogs, we used 5-nitrofuran-2-methylidene hydrazide and 5-nitrofuran-2-carbohydrazide substructures to query the MLSMR data set. The latter did not yield any analog, while the former resulted in 5 analogs, including the already described HC2250 (Data set 5). The analogs maintained a high inhibitory activity against the WT and the mutants (Data set 5, Figure 6).

##### 5-Nitrofuran-2-methanone Piperazinyl Benzothiazoles

Our statistical outlier analysis of the *mmpL3* mutant screen showed that the mutant pool exhibited enhanced sensitivity to some compounds (Figure 1B, Data set 2). These include seven analogs of nitrofuranyl/nitrothiophenyl benzothiazoles among others (Data set 5). In the structural similarity clustering of these analogs, two additional analogs (**977** and **1109**) were also identified (Figure 5, Data set 5). A substructure similarity search of the data set did not reveal any additional analogs, indicating that all the analogs are well represented in the cluster. A side-by-side comparison of the potency of the analogs against the WT and the mutants revealed informative trends (Figure 6, Data set 5). First, modifications at different positions of the benzothiazole ring did not impact the activities of the analogs against the WT. Second, all the analogs exhibited enhanced activity against the mixed *mmpL3* mutant pool. This collateral sensitivity also extended to the *katG* transposon mutant but does not extend to the *hadAB* mutant. Since the scaffold contains a nitro group that can easily form reactive species, we can speculate that the enhanced activity in the *katG* mutant background may be due to the absence of KatG, an oxidoreductase that normally removes toxic reactive oxygen species. The collateral sensitivity in the *mmpL3* background may be explained by the increased cellular entry of the compounds, although these hypotheses need to be tested. In any case, this scaffold may represent a component of future combination regimens that contain either isoniazid or MmpL3 inhibitors.

### Pks13 Inhibitors

**Figure 7:**
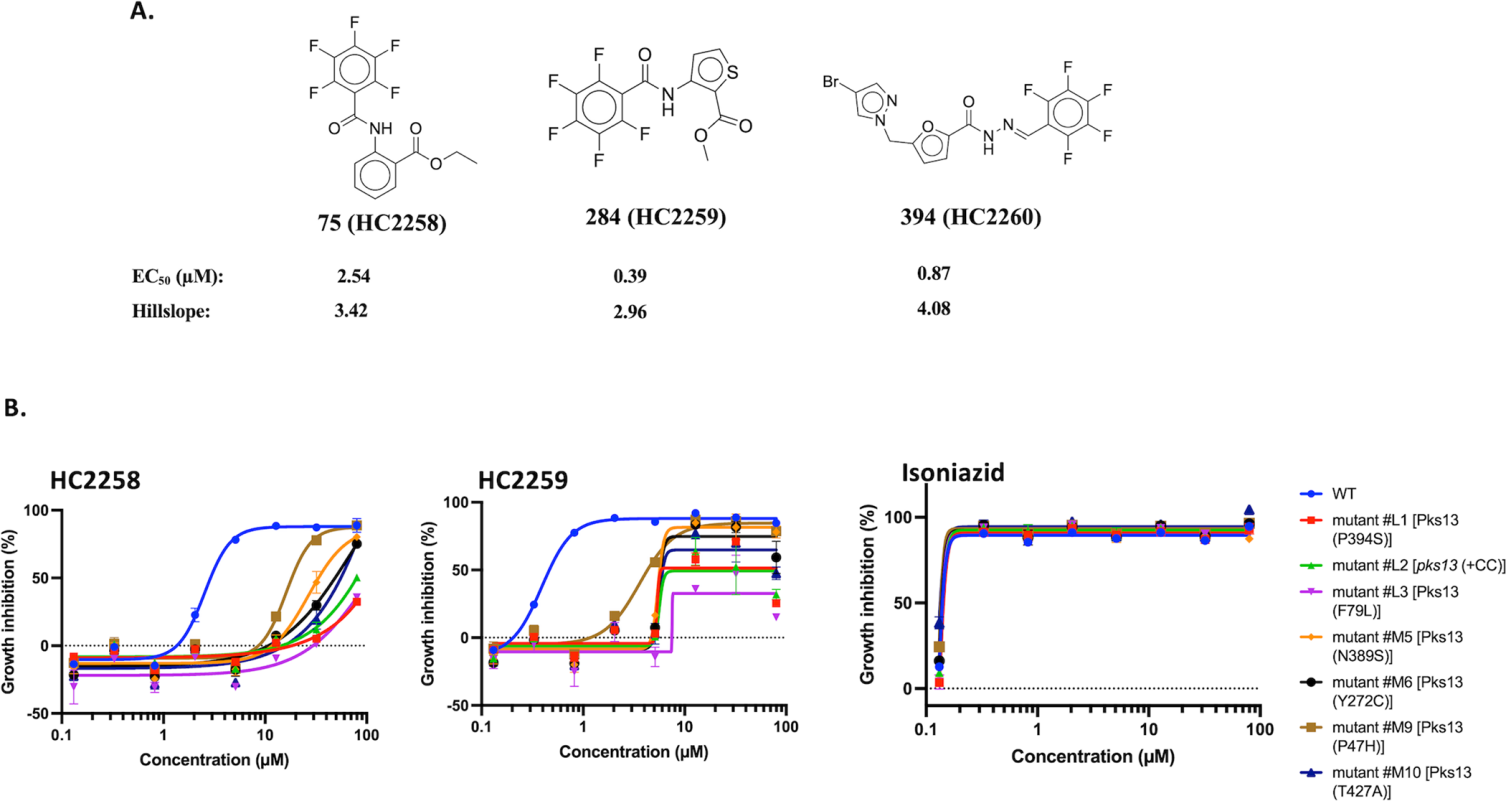
Identification of Pks13 inhibitors from follow-up studies. (A) Structures of the Pks13 inhibitors that were studied. (B) Cross-resistance screening of the *pks13* mutants. For the named mutants in panel (B), mutants labeled L (L1 and L2) had large colonies when selected, and mutants labeled M (M5, M6, M9, and M10) had medium sized colonies when selected. The mutant labeled + CC had an insertion of two nucleotides, disrupting the coding sequence.

One class of Pks13 inhibitors includes molecules that have a thiophene group linked to a pentafluorobenzyl carboxamate scaffold.^30,31^ Since Pks13 is an essential enzyme involved in mycolic acid biosynthesis, we explored the MLSMR Mtb inhibitors for other putative Pks13 inhibitors that have a pentafluorobenzyl carboxamate scaffold. When we queried our MLSMR Mtb inhibitor collection, only three compounds, **75**, **284**, and **904**, had this scaffold. However, when we used only pentafluorobenzyl as the structure query, we saw that a total of six compounds in our collection (**75**, **284**, **382**, **394**, **904**, and **1052**) had the substructure. To confirm if these compounds are Pks13 inhibitors, we purchased fresh powders of **75**, **284**, and **394**, renaming them HC2258, HC2259, and HC2260, respectively. In a dose–response study, we reconfirmed that these compounds are active against Mtb, with HC2259 being the most potent compound (Figure 7A). The potencies of the thiophenes in our study, HC2258 (EC_50_ = 2.54 μM; MIC_99_ = 9.74 μM), HC2259 (EC_50_ = 0.39 μM; MIC_99_ = 1.84 μM), and HC2260 (EC_50_ = 0.87 μM; MIC_99_ = 2.68 μM), are comparable to what has been reported for other antitubercular thiophenes where their MIC values ranged from 0.5 to 20.2 μM.^30^ We followed up our study by generating mutants that are resistant to HC2259 (Figure S4) and sequencing to confirm resistance. A relatively low frequency of resistance (1 × 10^–8^) was observed for HC2259, agreeing with what has been reported for other Pks13 inhibitors.^32^ Predictably, all of the resistant mutants had genetic changes in *pks13*, mostly point mutations, implicating the gene as a possible target of the compound. In a cross-resistance screen, all of the tested mutants were also resistant to HC2258 and HC2259, suggesting Pks13 as a shared common target (Figure 7B, Figure S4). Moreover, in agreement with previous studies,^30,32^ TB drugs such as isoniazid and ethambutol that target cell wall biosynthesis retained most of their activity against the mutants (Figure 7B, Figure S4), Overall, HC2258, HC2259, and HC2260 are putative Pks13 inhibitors, although biochemical data to this effect need to be provided.

## Conclusions

This study used a combination of genetic and cheminformatic tools to provide early mechanistic insight into the antimycobacterial activities of some compounds from the MLSMR library. These insights can guide further studies, especially using biochemical approaches, to confirm the mechanisms of action of these compounds. Our study provided a prioritization pipeline for some antimycobacterial hits from the MLSMR library. For instance, the isoniazid analogs that have a KatG-independent antimycobacterial activity need to be prioritized for possible development as TB drugs. The nitrofuranyl benzothiazoles have the possibility of being included in combination regimens for TB treatment with MmpL3 drugs such as SQ109 or the KatG-dependent drug, isoniazid. Additionally, the new compounds that we identified from our screen as putative MmpL3 or HadAB inhibitors can serve as training sets for machine learning possibilities in TB drug development. A limitation of this study is that the relative activities of the cherry-pick compounds, which have been subject to multiple freeze–thaw cycles, may not translate to what may be obtained using fresh powders. Additionally, without resynthesis and confirmation of the activity, it is possible that some chemical identities may be incorrect. Interpretation of the findings needs to be considered with this caveat, and resynthesis of key analogs is required prior to more extensive studies.

In recent years, artificial intelligence-based approaches are emerging for the discovery of new drugs.^33^ Machine learning algorithms are dependent on high-quality, feature-rich data sets on which to train models. It is our hope that the functional characterizations in our study can be used to enrich training models, and this resource will spur artificial intelligence-driven drug discovery and development for Mtb. Overall, this resource should serve as a valuable source of information for antimycobacterial compounds that can be studied to further understand mycobacterial physiology and develop new TB drugs.

## Materials and Methods

### Culture Conditions and Targeted High-Throughput Mutant Screening

Unless otherwise indicated, the different *Mycobacterium tuberculosis* (Mtb) strains used in this study were cultured and maintained in 100 mL 7H9 OADC with glycerol, Tween-80, and hygromycin, and the media was buffered to pH 7.0 with 100 mM MOPS. The cultures were allowed to grow at 37 °C in 5.0% CO_2_. Previously described methods were adapted in the targeted high throughput screening.^2,3,8^ Briefly described, the 935 cherry-pick hits from the MLSMR library were diluted 2.5-fold starting at 8 mM and used in an 8-dose response study to test the cultures. For the screening, Mtb CDC1551 *hspX*’::GFP reporter strain (WT) and the different mutants (*mmpL3* mutant pool; *hadAB* mutant; *Tn:KatG* mutant) were cultured to mid-log phase (OD_600_ ≈ 0.6) in 7H9 medium. This was followed by aliquoting 50 uL of the cultures into 384-well plates at an initial inoculum of OD_600_ = 0.05. Treatment was initiated by adding 0.5 μL of each compound, giving rise to a final concentration of 80–0.13 μM. DMSO and rifampicin were used as negative and positive controls, respectively. Plates were incubated with a wet paper towel for 6 days at 37 °C in 5% CO_2_ Incubator. Note that the reporter strain requires the addition of hygromycin in the medium to select for the plasmid, and no hygromycin was used in the mutant screens; otherwise, the screening conditions were identical for each strain. The absorbance (OD_600_) of the cultures was then read on a PerkinElmer plate reader, and the percent growth inhibition was calculated relative to controls. The area under the curve of the dose–response curve was used as a relative measure of potency and was calculated in GraphPad Prism (version 10). The Mahalanobis outlier method was used to identify outliers in the WT vs *hadAB* screen, as well as WT vs *mmpL3*, and this was done with the statistical package, SPSS.

### Eukaryotic Cytotoxicity Assay

Primary bone marrow-derived macrophages (BMDM) were obtained and cultured using a previously described protocol.^34^ This was followed by seeding 384-well opaque plates with the macrophage cells and treating them with different concentrations of the compounds as described in the targeted mutant screening above. DMSO and 4% triton X-100 were included as negative and positive controls, respectively. The macrophage plates were then incubated with a wet paper towel at 37 °C and 5% CO_2_. After 6 days of treatment, cell viability was assessed using the cell titer glow assay (Promega) and percent cytotoxicity was calculated relative to DMSO and 4% Triton X-100 controls. The area under the curve of the dose–response curve was calculated in GraphPad Prism (version 10).

### Intracellular Mtb Growth Inhibition

BMDM were obtained and seeded into 384-well opaque plates as previously described.^34^ After 24 h of seeding, the macrophages were infected with a Mtb CDC1551 strain expressing firefly luciferase at a multiplicity of infection of 1.^34^ Infection was allowed to proceed for 1 h at 37 °C followed by treatment with the compounds in a dose–response study as described above. After 6 days of treatment, the bright glow luciferin assay (Promega) protocol was used to assess the growth of the intracellular Mtb. Due to an edge effect, DMSO-treated cells could not be used as negative controls, and percent intracellular growth was instead measured relative to rifampicin and the average bacterial growth of Mtb treated with the lowest concentrations tested as the negative control.

### Similarity Clustering and Activity Cliff Analysis in DataWarrior

SDF files for each compound were provided by the NIH and were inputted into Datawarrior software.^15^ The Skelphere molecular descriptor of the compounds was calculated and used for clustering similar compounds in DataWarrior under the default settings. The Skelphere descriptor was also used in the activity cliff analysis, with the area under the curve of the compounds against the WT being used as a measure of their activity.

### Isolation and Characterization of Pks13-Resistant Mutants

The isolation and confirmation of resistant mutants were done as previously described.^8^ Briefly, 1 × 10^9^ CFU of CDC155 Mtb cultures was plated onto 7H10/OADC agar plates amended with HC2259. The plates were incubated at 37 °C until colonies appeared. The colonies were regrown in 7H9OADC and reconfirmed for resistance in a dose–response study. This was followed by whole-genome sequencing of the mutants and comparing the changes to those of the WT to identify the resistance gene.

### K-means Clustering and Hierarchical Clustering

K-means clusters were generated using the z-score standardized AUCs of each compound in each of the four *in vitro* conditions (WT, *mmpL3*, Tn:*katG*, and *hadAB*) using the *kmeans* function in R (Version 2024.09.0 + 375) (*k* = 8 based on elbow plot).^35^ Dendrograms were then generated by generating a distance matrix of the assigned k-means (1–8) for each compound using the *dist* function in R and clustered using the *hclust* function.^36^ To generate hierarchical clusters for compounds of similar function (e.g., INH-analogs) a structure similarity plot was first generated in DataWarrior (Version 5.5.0)^15^ based on *OrgFunctions*. XY coordinates for each compound were then extracted and used to generate a distance matrix using the *dist* function in R (method = Manhattan).^37^ Compounds were clustered using the *hclust* function in R using “average” linkage clustering to reduce outlier effects. Hierarchical clusters were compared to structure similarity plots in DataWarrior to ensure the reliability of the method. Both K-means and hierarchical cluster-based dendrograms were illustrated using the *pheatmap* function in R.

## Supporting Information Available

(Figure S1) Hypothetical 8-point dose–response curves relating calculated AUC, EC50, and MIC; (Figure S2) activity cliff analysis of the isoniazid analogs from the MLSMR data set; (Figure S3) pairwise structure–activity relationship study of some isoniazid analogs in the MLSMR collection; (Figure S4) Pks13 screening and cross-resistance studies (PDF)
(Data set 1) *In vivo* and *ex vivo* AUCs (XLSX)
(Data set 2) Analogs with resistance in *mmpL3* mutant mix (XLSX)
(Data set 3) Analogs with resistance in *hadAB* mutant mix(XLSX)
(Data set 4) Analogs with resistance in *katG::Tn* mutant(XLSX)
(Data set 5) Analogs containing nitro groups (XLSX)
(Database 1) Database file suitable for browsing compounds in DataWarrior (TXT)

## Abbreviations Used

AUC: area under the curve
BMDM: bone marrow-derived macrophage
EC_50_: half-maximal effective concentration
HTS: high-throughput screening
ISO: isoxyl
MLSMR: Molecular Libraries Small Molecule Repository
Mtb: *Mycobacterium tuberculosis*
SALI: structure–activity landscape index
SAR: structure–activity relationship
TAC: thioacetazone
TB: tuberculosis
WT: wild type
