# Supplementary material for: Genetic and Cheminformatic Characterization of Mycobacterium tuberculosis Inhibitors Discovered in the Molecular Libraries Small Molecule Repository
Source: ACS Infect Dis. 2025 Mar 25;11(4):882–93. doi: 10.1021/acsinfecdis.4c00936 (PMC11997997; doi:10.1021/acsinfecdis.4c00936)
Supplement: Supplementary file 1 — id4c00936_si_001.pdf [file id4c00936_si_001.pdf]

Supporting Information

Genetic and cheminformatic characterization of *Mycobacterium tuberculosis* inhibitors discovered in the Molecular Libraries Small Molecule Repository

Ifeanyichukwu E. Eke[1], John T. Williams[1], Robert B. Abramovitch*

Department of Microbiology, Genetics & Immunology, Michigan State University, East Lansing, Michigan, 48824, United States.

[1]*Authors contributed equally to this study.*

**Corresponding Author:*
Robert B. Abramovitch
E-mail: abramov5@msu.edu

Supplementary Figure 1. Hypothetical 8-point dose response curves relating calculated AUC, EC_{50} and MIC.

Supplementary Figure 2. Activity cliff analysis of the isoniazid analogs from the MLSMR dataset. The colors represent the AUC or activity of the analogs against the WT, while the size of the sphere indicates the value of the structure-activity landscape index (SALI). * = analogs that are compared in Supplementary Figure 3.

Supplementary Figure 3. Pairwise structure-activity relationship study of some isoniazid analogs in the MLSMR collection. Delta activity is the difference in the AUC values of the two compounds against the WT culture of Mycobacterium tuberculosis.

Supplementary Figure 4. Pks13 screening and cross-resistance studies. A. Confirmation of HC2259-resistant mutants. **B.** Cross-resistance screening of the mutants against HC2260 and ethambutol.

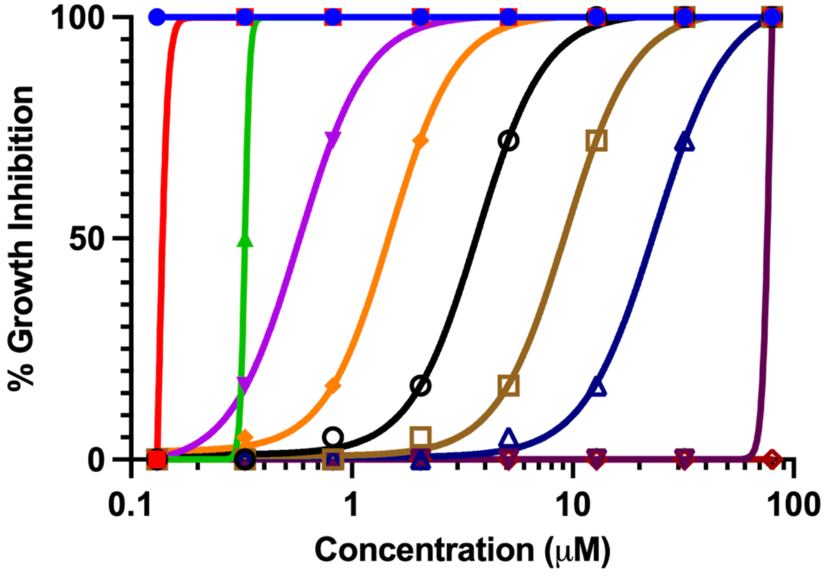

		μM	
	**A.U.C.**	**EC_{50}**	**MIC**
● (blue)	278.6	N.D.	N.D.
■ (red)	258.7	0.1347	0.182
▲ (green)	238.8	0.3277	0.4632
▼ (purple)	214.4	0.5996	1.189
◆ (orange)	176.6	1.476	2.933
○ (black)	136.8	3.809	7.362
□ (brown)	97.02	9.281	18.44
△ (navy)	57.22	23.94	46.15
▽ (dark purple)	19.90	77.87	81.28
◇ (dark red)	0	N.D.	N.D.

Supplementary Figure 1

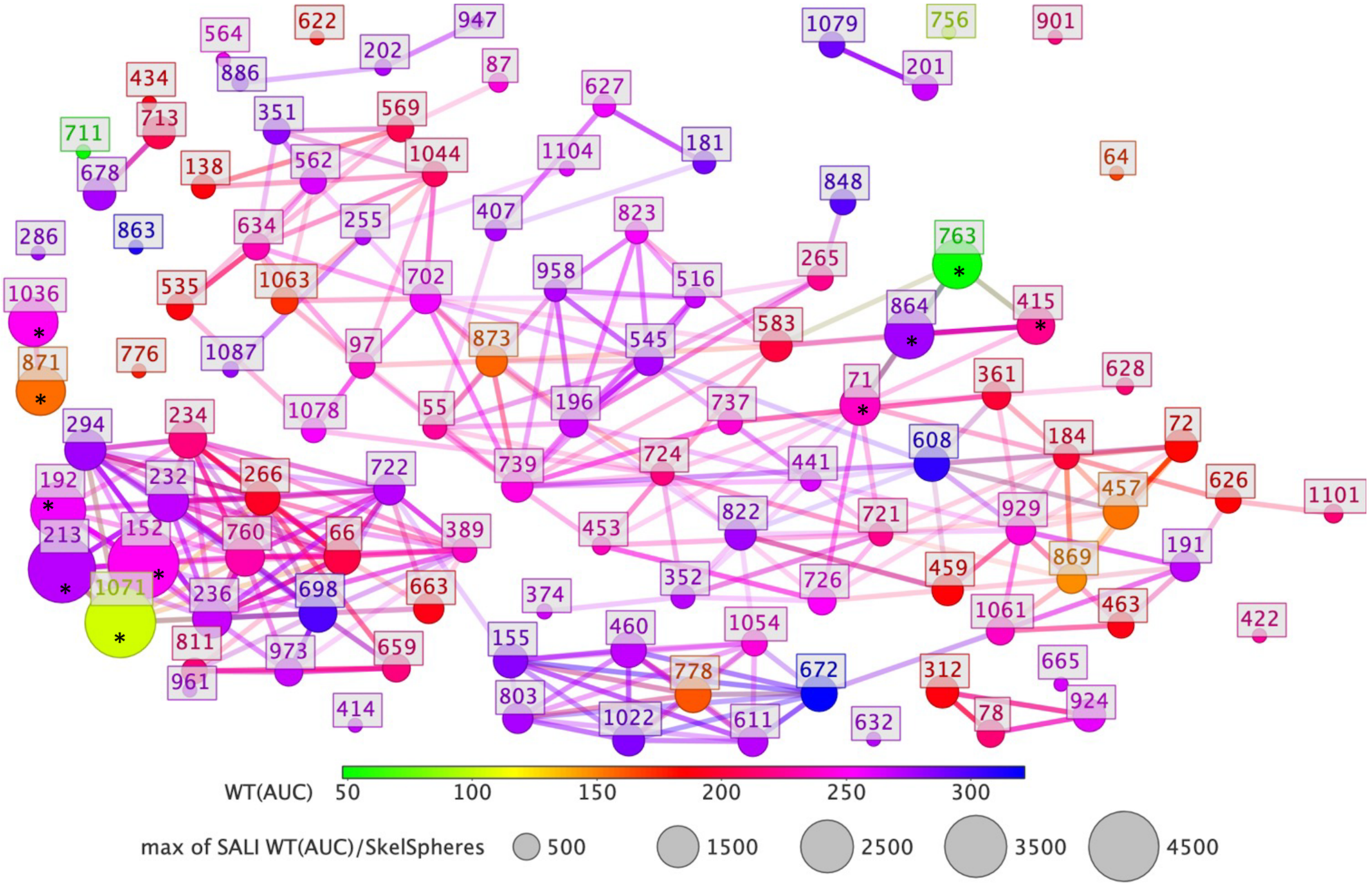

Supplementary Figure 2

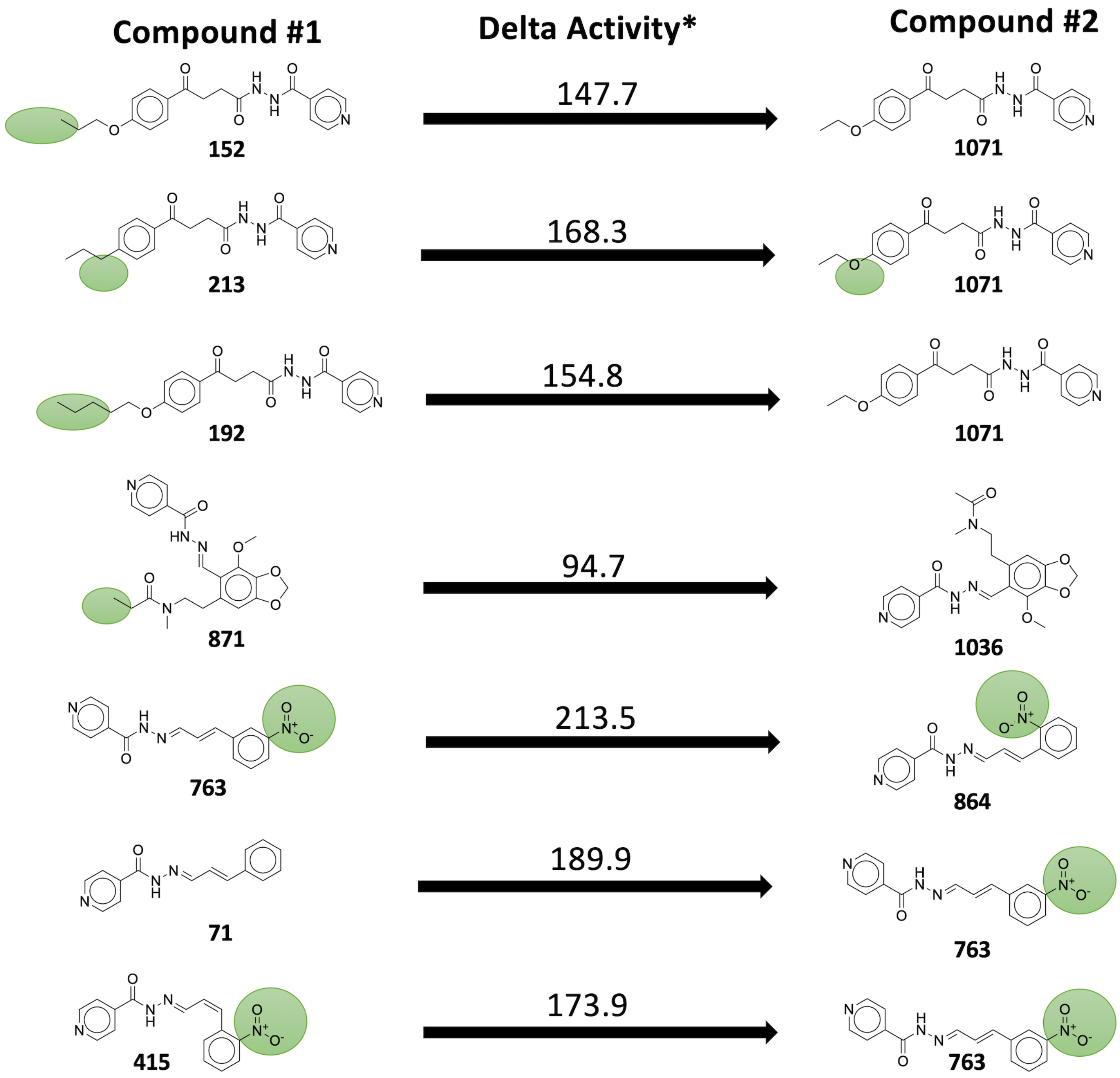

Supplementary Figure 3

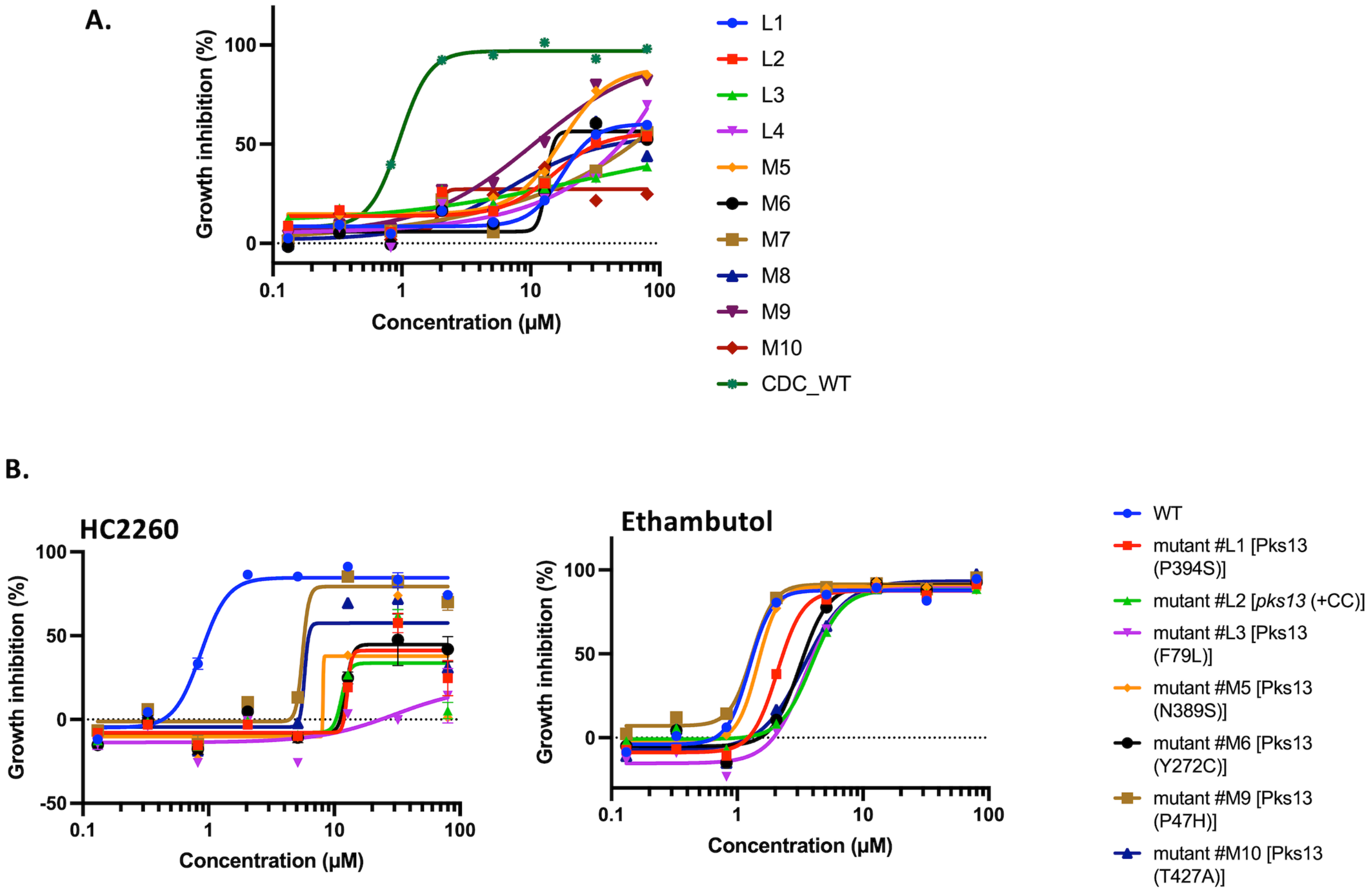

Supplementary Figure 4
